# Psychology Education and Work Readiness Integration: A Call for Research in Australia

**DOI:** 10.3389/fpsyg.2021.623353

**Published:** 2021-04-09

**Authors:** Ashleigh Schweinsberg, Matthew E. Mundy, Kyle R. Dyer, Filia Garivaldis

**Affiliations:** ^1^School of Psychological Sciences, Monash University, Melbourne, VIC, Australia; ^2^Monash Centre for Professional Development and Monash Online Education, Monash University, Melbourne, VIC, Australia; ^3^Institute of Psychiatry, Psychology and Neuroscience, King’s College London, London, United Kingdom; ^4^Monash Sustainable Development Institute, Monash University, Melbourne, VIC, Australia

**Keywords:** work readiness, psychology workforce, Australia, graduate attribute, pedagogy of psychology, psychology

## Abstract

Supporting students to develop transferable skills and gain employment is a vital function of Universities in the era of the Fourth Industrial Revolution. A key area is work readiness, which has steadily grown in importance over the last 2 decades as tertiary institutions increasingly aim to produce graduates who perceive and are perceived as *work ready*. However, a large majority of graduates report a lack of skills and confidence needed for the effective transition from study to work. This may be particularly problematic for disciplines that impart both discipline-specific and transferrable skills, such as psychology. The aim of this paper is to addresses the concept of work readiness within Australian psychological training and explores the need to shed light on and integrate work readiness within the pedagogy of psychology within Australia. Specifically, this paper calls for a review of work readiness skills developed in psychological courses to ensure industry needs are met. Beyond such a review, it is suggested that tertiary centres need to facilitate students in capturing and reflecting upon the transferable skills that they develop; and build assessments that allow students to demonstrate transferable skills in a meaningful way. Further, this paper proposes that work readiness skills be routinely mapped onto graduate attributes and course learning outcomes to be readily available by students so as to increase students’ potential to articulate their learnt work readiness skills once in the workplace.

## Introduction

### Background and Context

In 2021, university funding within Australia will likely be focused on areas deemed to have critical skill shortages, such as those requiring specialist knowledge (Department of Jobs and Small Business, 2018; Australian Government, 2020b). Indeed, in a skills-based economy, and particularly in the volatile job market post-2020, graduates require intimate knowledge and understanding of how to inform future employers of their acquired skillsets (World Economic Forum, 2019). The need to articulate, capture and reflect on the variety of skills that are learnt during a university course is central to a successful transition from study to career, especially in the mental health arena. The skills of graduates are particularly important in the mental health field where pre-existing shortfalls, gaps, and discrepancies between skills acquired during study, and skills required at employment are widening, whilst the need for mental health professionals is growing (Gardner and Liu, 1997; Casner-Lotto and Barrington, 2006; Australian Institute of Health and Welfare, 2019; Black Dog Institute, 2020). Indeed, tertiary graduates are expected to enter the workforce as “work ready” (Clarke, 2018). There is an onus on both tertiary institutions and the student themselves when considering this topic. It is the institution’s responsibility to make available information and the student’s responsibility to absorb such information. For instance, according to the Australian Grattan Institute report of 2008 mapping higher education, “*most higher education students are seeking vocational outcomes*” (Norton and Cherastidtham, 2018, p. 10). In the field of psychology, in Australia and many other nations, discipline specific skills are typically addressed predominately during postgraduate education. Such course structure may impact the perception of work ready courses which may in an Australian context, determine tertiary funding in the future. However, this paper will look predominately at the undergraduate psychology stream.

The ability to understand and describe skills that students learn during their tertiary education is growing in importance particularly in Australia with the adoption of the Bologna Process, which is intended to enable Australian award graduates greater international portability between European and Oceania nations (Jackson, 2019; Department of Education Skills and Employment, 2021). Fuelling this debate are calls from the industry base to better understand the skills imbued to graduates through tertiary programs with a recent review by Prikshat et al. (2020) reporting significant perceived deficits in Australian graduates understanding, which is steadily increasing in prevalence. The concept of graduate work readiness has emerged in the literature base in the last 2 decades with journals, such as Education + Training sponsoring special editions to focus exclusively on the topic (Turner and Winterton, 2019). Such focused attention is revealing a need to identify not only generic skills, which contribute to graduates’ employability, but also discipline-specific skills, which are unique to each industry steam. The combination of generic, discipline-specific, meta-competencies, hard (measurable) and soft (interpersonal) skills can be captured in the term *work readiness*. In other words, graduates who are work ready must possess a range of skills that enable them to excel in tasks that are common to all industries, as well as unique to their specific industry – ultimately making them able to enter their trained in discipline.^1^ Similarly, disciplines themselves are required to respond to requirements of the professions they train for to ensure they maintain fidelity as well as relativity (Prikshat et al., 2020). Whilst the discipline of psychology is well known for the transferrable skills that it imparts on students, and which contribute to their overall employability, the extent to which specific and non-transferrable skills are attained in psychology education has not yet been explored, particularly in an Australian context (see Jackson, 2019). This paper will focus on the discipline specific skills of psychology. Specifically, this paper aims to articulate the gap in the literature base within the pedagogy of Australian psychology.

### Aims and Scope

As described, work readiness is comprised of generic and discipline-specific personally orientated skills and traits possessed by a graduate and reflect the level of preparedness for successful transition from student to employee (Caballero and Walker, 2010). Furthermore, recent research has called for both occupation specific and country contextualised work readiness research, as it is argued that work readiness is not homogeneous (Tempone et al., 2012; Winterton and Tuner, 2019). Therefore, defining the discipline-specificity of work readiness in all disciplines, including psychology, is critical. Work readiness skills predict job performance, career advancement, and even potential for promotion, all of which are context (discipline) specific (Gardner and Liu, 1997; Crebert et al., 2004; Hart, 2008). Finally, work readiness helps shape graduates’ professional identity, which is a concept that manifests differently across disciplines and career pathways (Hind et al., 2003; Day and Tytler, 2012; Winterton and Tuner, 2019). As such, discipline specific research into work readiness may assist graduate outcomes.

As work readiness is in its infancy within the discipline of psychology, it is premature for a systematic literature review to occur until the area has matured. As recently as last year, Abelha et al. (2020) completed a systematic review into how higher education institutions prepare gradates for professional practice and found only 69 papers world-wide addressing the topic. Narrowing down further, only seven articles were found to reference the United Kingdom and Australia, and only three papers in the field of psychology, pedagogy, and education. Importantly, the Abelha et al. (2020) review highlights the growing competence-based nature of higher education.

An argument could be made that an overview of work readiness for psychology is not required as research has been completed into the broader health professions, such as nursing (Aznal et al., 2019; Baumann et al., 2019). However, psychology is a distinct and separate discipline, and limited published research currently exists as to how psychology is differentiated from other allied health professions, such as mental health nursing, occupational therapy, or social work. Adopting the definition that work readiness is both discipline specific and generic, the purpose of this paper is to focus on the disciplinary specific attributes of psychology, which are recognised by registration bodies such as the Australian Health Practitioner Regulation Authority (APHRA) and the Australian Psychological Society (APS). The current paper discusses how graduate attributes instilled in psychology degrees in an Australian context can develop students’ work readiness. Suggestions will be made as to how the link between graduate attributes and on-the-job demands can be strengthened and better understood especially in the area of perception of graduates in acknowledging the skills learnt during their university career. We propose that the work readiness of psychology graduates needs to be addressed both at the undergraduate as well as the postgraduate level of education, so that graduates of all degree levels, can be work ready (Guthrie, 2016). Firstly, there will be a discussion concerning psychology education in Australia, followed by a brief exploration of potential skill mapping and linking graduate attributes to work skills.

## How Psychology Education in Australia Prepares Students

Between undergraduate and postgraduate study there is a significant attrition of students (Bryan et al., 2010). Whilst publicly available data is scarce, it is estimated that over 60% of students do not progress to further study; however, such figures need to be used with caution as they are part of aggregated data with other health related courses (Guthrie, 2016). Today, registered with Australian Psychology Accreditation Council (APAC) there are 317 undergraduate compared to 131 postgraduate accredited degree programs (APAC, 2020). The narrowing of the candidate field is deliberate and continues as students’ progress with the Australian Qualification Framework. As such, the question exists as to what knowledge a graduate of a psychology undergraduate program possesses and where do they apply their perceived skill sets.

Ultimately, the primary aim of psychology courses is to prepare students to be a psychologist. Indeed, all courses require accreditation and follow a carefully curated program. Importantly, undergraduate programs are knowledge based whereas postgraduate programs are skill and vocationally based (Tee et al., 2018). However, there is not clear delineation between skills obtained in undergraduate compared to postgraduate study. In Australia, the Carrick Institute developed a framework for psychological accreditation and learning overseen by the APAC. APAC’s role is to ensure that “*graduates of accredited programs receive high-quality education and are well equipped to employ their psychological knowledge and skills in the community*” (APAC, 2019). The APAC framework introduced in 2009 nationalised the psychology profession and aimed for psychology graduates to possess a common set of skills, such as industry-specific academic knowledge and clinically orientated skills, which theoretically ease transition into the workforce (Carrick Associate Fellowship Project, 2008). Such efforts include the introduction of the National Psychology Examination (Carrick Associate Fellowship Project, 2008). The Carrick review called for regular reviews of psychological attributes; however, such a review appears overdue.

### Pathway to Becoming a Psychologist

**Figure 1:**
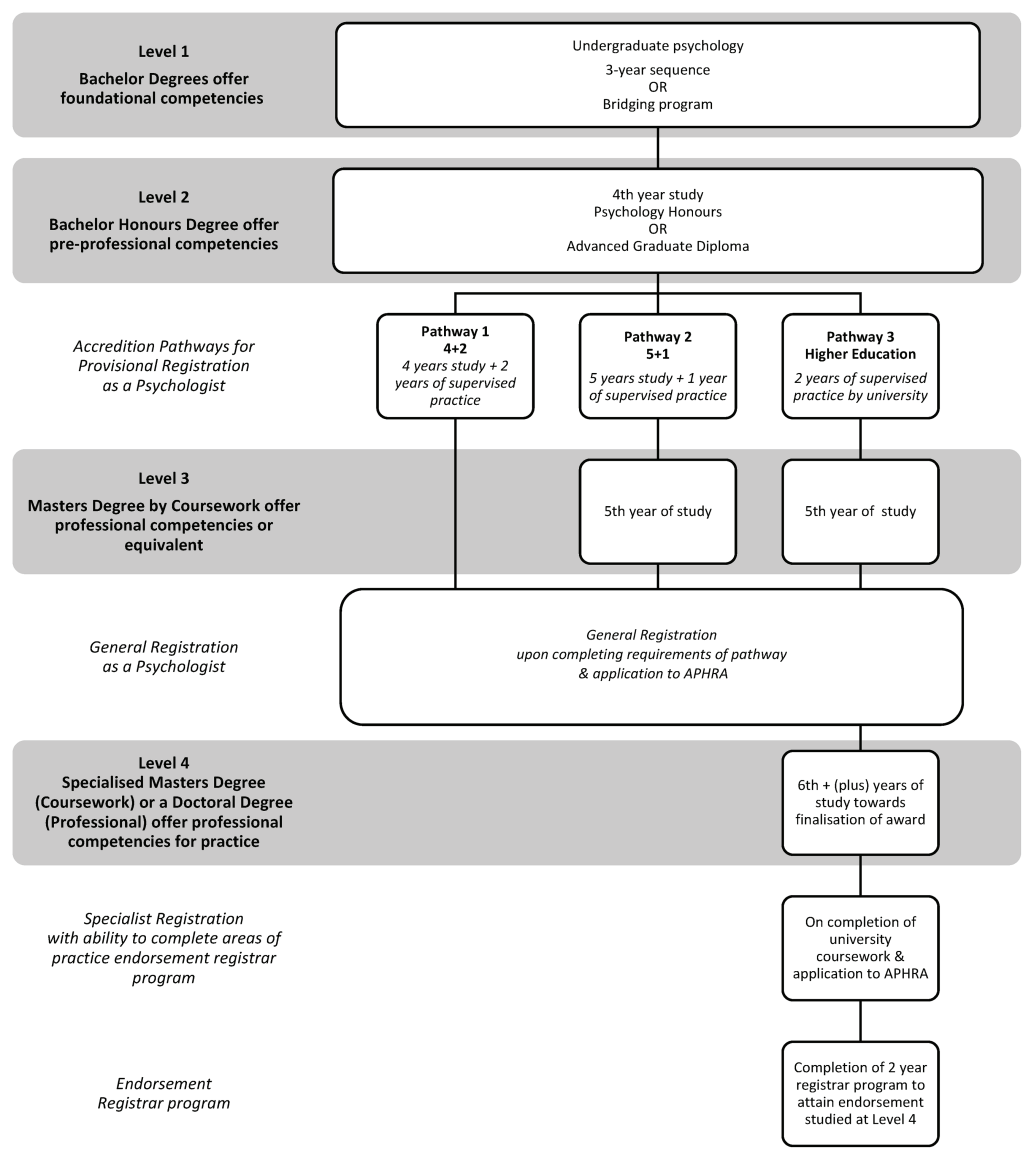
Australian Psychology Accreditation Council (APAC) accreditation pathways for registration as a psychologist in Australia. Adapted from: APAC (2019, 2020). Please note general bridging courses are being developed and approved for release as of July 2019 for Pathway 1 and 2. Pathway 1 will be retired as of 2029.

To understand where gaps in graduate knowledge may occur, an exploration of what is intended to be learnt during psychology training, which is geared towards enabling registration needs to occur. The psychology profession relies on discipline-specific skills being attained *via* a university degree. Graduates of a 3-year undergraduate sequence in psychology graduate with *foundational competencies*, which are the foundation and knowledge development for psychological practice, as per the scientist-practitioner model (Geffen, 1993). At this level of professional training, students are not yet explicitly instructed in how to apply or use acquired knowledge within a professional context. Indeed, some scholars argue that undergraduate psychology courses should remain focused only on knowledge acquisition (Cranney et al., 2016). However, once graduates progress to post-graduate studies, the focus reorientates to practical skill building, be it for a career in research, clinical practice, or other industries. APAC prescribes the available registration pathways, which permit graduates to attain registration as a psychologist and are the underpinnings of all psychology courses at Australian universities. Accreditation is level based, commencing with *foundational competencies* as described above at level one and progressing through to *professional competencies for practice* at level four. Further detail can be seen in Figure 1, which displays these registration pathways, as well as the expectations of competence at each level.

### Alternative Job Pathways of Psychology Graduates

Most critical, for students not pursuing registration as a psychologist, there appears to be insufficient evidence as to where and how alternative career paths allow them to utilise and apply knowledge gained during their psychology education. Whilst general information on graduate career pathways is available from various data sources, such as the Graduate Outcomes Survey (The Social Research Centre, 2020); psychology undergraduate courses are considered to form part of the Australian Bureau of Statistics “society and culture” category, which includes “social sciences,” “behavioural science,” and “philosophy and religious studies” (ABS, 2001). Additionally, at an institution level, multiple faculties and schools administer psychology subjects (Karp, 2020). This makes understanding the specific outcomes of psychology graduates more complicated.

Indeed, the lack of differentiation of psychology subjects at undergraduate level has been highlighted in recent government initiated tertiary fee reforms. In June 2020, it was made public that as of 2021, post-graduate Clinical Psychology course fees will be reduced, whilst undergraduate courses will significantly increase (ABC News Live, 2020b). However, due to significant public lobbying it was announced that psychology courses fees would not be increased (Hunter, 2020). Perhaps, we will have a clearer picture of psychology graduate outcomes should the discipline be separated into its own category (ABC NEWS Live, 2020a; Hunter, 2020). Despite lack of clear differentiation, psychology subjects are valued for their ability to develop transferable skills and broad learning of human behaviour. The debate is not concerning whether graduates are learning skills, but rather what skills are learnt and when. Thus, the question at hand is one of recognition of current practice and pedagogy.

## The Question of Transferable Skills

The APS suggests that university qualifications in psychology allow graduates to pursue careers in fields such as child protection services, law enforcement, and academia (APS, 2020). Other internet sources targeted at undergraduates suggest that potential career paths include media analyst, teacher, and writer (Mallory, 2020). Identified transferable skills include, understanding human behaviour, research, and critical thinking, with commonality found in clear communication and close interactions with others. For instance, understanding and harnessing human motivation aids development of strong advertising campaigns. According to the Australian Government (2020a) Job Outlook data, the top five skill-sets expected of psychologists in an Australian context, in descending order of perceived importance are: social perceptiveness, active listening, reading/comprehension, speaking, and active learning.

Given that such a significant proportion of students cease studying psychology following the first 3 years of study, questions arise as to what transferrable skills are learnt during these first 3 years, and which skills can be used in alternative industries (APAC, 2020). Widescale Australian research suggests that psychology graduates spend over 2 years in the hospitality trade before formally entering the psychology profession (Carroll, 2013; Guthrie, 2015). As such, and irrespective of whether graduates select to pursue the academic or professional pathway, students and graduates of psychology experience challenges in the transition towards professional vocation. Having accessible, interpretable and cross sector usability and understanding of skills may enable psychology gradates to better understand what they have learnt and where they can apply it. To date, such data are not available. Having such data may advance gradate outcomes as well as align tertiary education with the unfolding fourth industrial revolution (for more information, see Schwab, 2016; Australian Government, 2021). Therefore, focused research is required to allow elucidation of career and further studies undertaken by graduates of psychology courses, so as to better understand this skill set and where it can be applied in broader society.

Looking to the wider educational arena, Hamilton et al. (2018) call for the tertiary education sector to equip students to step away from study and develop “*psychological literacy*,” as coined by Boneau (1990). Prior to this, Cranney and Dunn (2011) suggested that psychological literacy affords individuals the ability to apply the practice of psychological science to societal, professional, and personal goals, and is not limited to the fields of mental health. Goedeke and Gibson (2011) went as far as to suggest that universities have an obligation to ensure that graduates of a 3-year degree sequence have wider career prospects than just the registration process. Perhaps the focus for accreditation of undergraduate degrees requires a shift to the broader application of the valuable transferable skills attained in the study of psychology rather than solely for professional registration. It is here that work readiness may play a role to assist easing the stress inherent in career transition.

The Carrick Associate Fellowship Project (2008) commenced and created a foundational base for attributes of graduates of psychology, which underpin course development (see Schweinsberg and Garivaldis, 2020 for further information). However, no further publications or publicly available research appear in the area of graduate psychology skills and how, or if, these are developed during tertiary education. For instance, the Project named six attributes, which should be instilled at the undergraduate level of training underpinning psychology education. These attributes commence with knowledge and understanding of psychology, progressing to research methods and critical thinking skills; followed by values and communication skills; with a capstone learning and application of a psychology project (Psychology Board of Australia, 2020). Such skills are in high demand across industry sectors, as evidenced through reviewing graduate attributes among different degrees within Australia (Karp, 2020). That said, there does not appear to be data relating to how psychology graduates are received by future employers with respect to the skills they possess. Further, the skills listed in Job Outlook are sourced from a United States database, which has been adapted for Australian use and may not have cross cultural validity (O’Net OnLine, 2019).

## The Need for Work Readiness for Psychology

When considering the need for work readiness specific to psychology, it has thus far been assumed that the discipline of psychology is not distinguishable from other allied health professions, such as occupational therapy, mental health care nursing, and social work. If this assumption is true, it would invalidate the separation of degree streams of psychological science. Through careful exploration of the discipline of psychology, specific skills developed through studying psychology can be defined (e.g., effective communication, research ability, and interpersonal effectiveness) and as such, the psychology profession can clearly position themselves as unique, separate, and valuable, both in terms of education and contribution to the wider working community.

In the field of psychology, work readiness research is sorely lacking and represents a significant gap in the literature. More interest is being generated, as can be seen by more recent papers on the topic, such as research by Hamilton et al. (2018). Turning to sister disciplines, growing research in the subject areas of emergency medicine (Edwards, 2011), nursing (Walker et al., 2013; Missen et al., 2015), and engineering (Crebert et al., 2004; O’Brien et al., 2012) has occurred in the last decade. To this end, we can see developments occurring in the field of work readiness, with discipline-specific scales being developed, such as the one by Walker et al. (2015) for nurses, to identify how work ready students are for particular disciplines. Walker et al. (2015) extended research commenced by Caballero et al. (2011) into the development of a work readiness scale that was discipline specific containing four factors: organisational acumen, work competence, personal work characteristics, and social intelligence. The nature of these factors suggests that such a scale of measurement may be applicable to the psychology profession, orientated to the unique skills of psychology graduates. Of note, a measurement scale applicable to a United States psychology population was developed in 2018 by Ciarocco and Strohmetz (2018) in conjunction with the American Psychological Society focused on the variable of self-efficacy. Such a scale could be applicable to an Australian base following systematic qualitative and quantitative research to complete gap mapping projects within the Australian population base.

### Linking Graduate Attributes to Work Readiness Skills

Graduate attributes are the basis of work readiness skills and form the basis of psychological competence. Psychology students and graduates have historically had difficulties drawing connections between the content of their degree and future use in employment contexts (Borden and Rajecki, 2000). Understanding work readiness may be the key graduates require to understanding the knowledge they have and how it can be applied to future employment, recalling this is a science of perception on behalf of the graduate.

The skills attained during tertiary education are partly defined by the graduate attributes of the completed course. Looking to educational counterparts at lower levels of the Australian Qualifications Framework, within TAFEs, for example, the link between graduate attributes and skills is clear. The framework is competency based, similar to that seen within a nursing framework (Health Workforce Australia, 2014). Such a clear linking of graduate attributes to skills is often not seen within psychology degrees and it is left to the students and future employers to surmise the link between the degree an employee candidate has undertaken and the role they are applying for.

**Figure 2:**
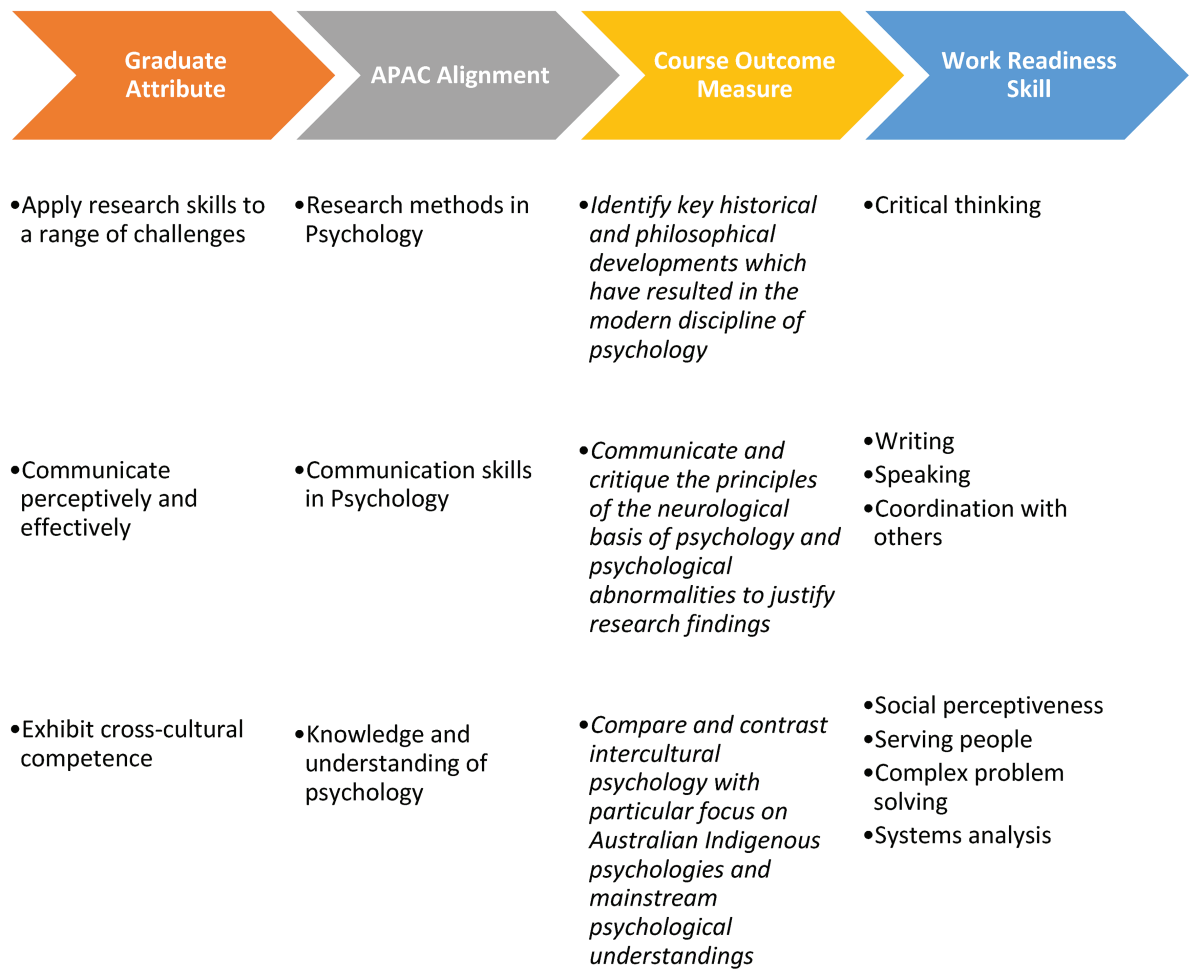
Example course mapping for first year introduction to psychologyundergraduate course. Information sourced from: Course outcome model (Monash University 2019 PSY1011 – Psychology 1A); Skill (Table 1); APAC alignment (APAC); Graduate attribute (Monash University Graduate Attributes – general).

It is proposed that the link between graduate attributes and skillsets should be tangible and easily accessible by students and employers through graphic displays linking a skill to the broader educational underpinning that coursework resides. Potential course mapping projects could use skill mapping between graduate attributes, industry attributes, work readiness skills, and course outcomes to show such skill development. Such images could be generated for all units and course outcomes and attached to learning modules and online course content for easy access by students. Whilst such data may be accessible to course convenors/creators, such data is rarely accessible by students and/or publicly accessible. It is the view of the authors that such meta views of the course are often inaccessible and non-understandable by students. By breaking a course down by learning outcome, which are already mapped to assignments, lectures, and tutorials would allow students to more readily and regularly see the skills that they are developing in the moment, allowing the opportunity for greater knowledge and understanding of the purpose of academic tasks undertaken. One such example can be seen in the graphic displayed in Figure 2. The concept of breaking down and defining key concept linking originates from the discipline of psychology itself and are taught during therapies such as Acceptance and Commitment Therapy or Dialectical Behaviour Therapy.

Further research into the skills developed by psychology graduates is required to provide the data for such an endeavour. Of note is that such endeavours have previously occurred in disciplines such as engineering (Student Futures, 2020) and Speech Pathology (Speech Pathology Australia, 2017), where tertiary courses closely align with industry needs. This has permitted each discipline to solidify their place in the modern industrialised and business activity led world.

## Conclusion

To date there has been an underinvestment in research pertaining to work readiness, particularly in the field of psychology in Australia, which represents a significant gap in the literature. Given that, we are approaching the 10-year anniversary of a major change in psychological education as commenced by the Carrick review, and current underway reviews into public funding for access to psychology treatment, such research is required to ensure psychology remains pertinent to a modern industry-based society (i.e., the fourth industrial revolution). Investigations into work readiness within psychology, at all levels of tertiary study would permit exercises such as skill mapping and transferable skill table development, as well as increase the potential adaptability and relevance of psychology orientated degrees. Importantly, such an endeavour is a first step permitting development of additional tools that cannot yet be explored without foundational research. Furthermore, such exploration will ensure that graduates of Australian degrees have greater international and cross boarder portability of studies as put forward by the Bologna process. Exploring the area of work readiness will elucidate rich information to the advancement of the scientific practice of psychology, such as reviewing the Carrick Institute recommendations in light of the previous decade and the numerous changes to both the psychology profession and the Australian culture. Adopting the stance that work readiness is both discipline specific and general, the purpose of this paper was to highlight the need for exploration of the *discipline specific* attributes of psychology graduates which would solidify the differentiation and uniqueness of the same profession. Further, such investigation would allow clear delineation of the skills that psychology graduates possess, which recognised by bodies such as APHRA and the APS are not understood or readily available by the general public or the industry base that hires graduates. Further, internationally, as commenced by the Bologna Process, understanding work readiness of graduates will permit international applicability and transferability of Australian degrees (Prikshat et al., 2020; Department of Education Skills and Employment, 2021). Specifically, this work advances the cause of investigating work readiness within the confines of the discipline of psychology. This is the start of the conversation, not the end.

Understanding the nuances of how psychology students navigate the transition between studying and being gainfully employed is central to workforce development. Firstly, it allows for greater comprehension and understanding of how to aid students to transition effectively from higher education and secondly it ensures that adequate numbers of psychology graduates enter the workforce (Victorian Government, 2018; Australian Institute of Health and Welfare, 2019; Australian Government, 2020a). Being able to articulate specific skills and thus understand their own work readiness, allows graduates to effectively inform future employers of their potential value to the host organisation. Further, increasing graduate’s knowledge and perception of skills learnt is likely to also increase their confidence in their own work readiness (recalling this is a matter of individual perception as opposed to a difference in *attained* knowledge). There is growing impetus to reconceptualise how work readiness is seen in the field of psychology so that effective, well rounded, empowered graduates enter the workforce. In a post, COVID-19 environment not only is psychology knowledge vital, so too will be a broader understanding of human behaviour to allow conceptualising of society, work, and education roles, which are rapidly changing. Work readiness is a marathon not a sprint and needs to occur from entry to tertiary education right up until final graduation, instead of being an afterthought triggered as graduation looms.

## Data Availability Statement

The original contributions presented in the study are included in the article/supplementary material, further inquiries can be directed to the corresponding author.

## Author Contributions

This manuscript was developed by AS under the supervision of FG and MM. AS led the writing of the manuscript with input from FG, MM, and KD. FG and MM contributed through expertise in direction, theory and manuscript review. KD contributed through overarching theory and final draft review. All authors contributed to the article and approved the submitted version

### Conflict of Interest

The authors declare that the research was conducted in the absence of any commercial or financial relationships that could be construed as a potential conflict of interest.

## Footnotes

^1^*As an example, Joe Bloggs may be well suited for field A, and have transferable skills to field B but would require training in the discipline specific skills of field B before being considered “work ready.”*
